# Short- But Not Long-Term Effects of Creep Feeding Provided to Suckling Piglets

**DOI:** 10.3390/ani15020253

**Published:** 2025-01-17

**Authors:** María Romero, Ana Heras-Molina, María Muñoz, Luis Calvo, José Ignacio Morales, Ana Isabel Rodríguez, Rosa Escudero, Clemente López-Bote, Cristina Óvilo, Álvaro Olivares

**Affiliations:** 1 Departamento de Producción Animal, Facultad de Veterinaria, Universidad Complutense, Avda. Puerta de Hierro s/n, 28040 Madrid, Spain; maria.romero@copiso.com (M.R.); andelash@ucm.es (A.H.-M.); rmescude@ucm.es (R.E.); clemente@ucm.es (C.L.-B.); 2 COPISO, Avda. de Valladolid, 105, 42005 Castilla y León, Spain; ji.morales@copiso.com; 3 Departamento de Mejora Genética Animal, Instituto Nacional de Investigación y Tecnología Agraria y Alimentaria, INIA, CSIC, Ctra Coruña km 7.5, 28040 Madrid, Spain; mariamm@inia.csic.es (M.M.); ovilo@inia.csic.es (C.Ó.); 4 Incarlopsa, Ctra. N-400, Km. 95.4, 16400 Castilla La Mancha, Spain; luiscalvo@incarlopsa.es (L.C.); airodrguez@incarlopsa.es (A.I.R.)

**Keywords:** piglet, creep feeding, microbiome, birth weight

## Abstract

Fifty-eight litters (16 from primiparous gilts and 42 from multiparous sows) were used, with a total number of 750 piglets involved in the study. Birth weight was stratified into three groups: low (<1.02 kg; LBW), normal (1.02–1.62 kg; NBW), and high (>1.62 kg; HBW). A creep feeding diet was offered to piglets in a creep feeder in 29 litters from day 7 until their weaning. Piglet mortality was recorded daily. Traceability was ensured up to the point of carcass splitting and subsequent meat analysis. Each carcass was eviscerated and weighed individually. Sixty-nine piglets were selected for the microbiome analysis (35 from the control group and 34 from the creep feeding group). Feces samples from the rectum were obtained at three time points (three days prior weaning, a week after weaning, and before the slaughtering of the pigs). Mortality during lactation was influenced by birth weight, with LBW piglets exhibiting a six-fold higher mortality rate than HBW. Creep feeding did not impact piglet mortality. Heavier piglets demonstrated greater weight gain when subjected to creep feeding, while the growth potential of lighter piglets was reduced. Variation in creep feeding consumption based on birth weight also affected microbiome composition, with high-birth-weight piglets displaying higher alpha diversity than low- and normal-birth-weight piglets seven days after lactation. Alpha diversity is indicative of gut health, with higher values suggesting greater stability and adaptability to different feed sources. In conclusion, the immediate impacts of creep feeding appear to be most prominent during lactation and potentially early postweaning. These short-term effects are modulated by birth weight, with HBW piglets demonstrating the greatest benefits from the implementation of creep-feeding practices.

## Simple Summary

Creep feeding is a common management tool on pig farms designed to encourage dry feed intake and supply piglets with essential nutrients by providing a highly palatable and easily digestible diet. Currently, research results on the efficacy of creep feeding vary, possibly because numerous factors may influence its benefits during lactation. Therefore, the aim of this study was to gain a deeper insight into the consequences of creep feeding on short- and long-term growth, carcass quality, meat characteristics, and microbiome composition as a function of piglet birth weight. During the study, we did not observe any long-term effects of creep feeding on these parameters. The short-term impacts of this nutritional management technique were mainly concentrated on lactation and potentially in the early postweaning stages. These short-term effects were influenced by birth weight, as piglets with higher birth weights seemed to benefit more from creep-feeding practices.

## 1. Introduction

Increase in prolificacy over recent years has boosted the number of low-birth-weight (LBW) piglets drastically, reaching proportions near to 15–20% [1,2]. This implies an increase in neonatal deaths and in the number of pigs with immature digestive capabilities and retarded growth [3,4]. Consequently, there is a high variability in batches along weaning and fattening, with concomitant economic losses, as well as a decrease in animal welfare [5,6].

Different handling strategies such as early management intervention of piglets and the promotion of colostrum intake has proven effective during the first days of life with the purpose of ameliorating piglet survival and further development [7,8,9]. Romero et al. [10,11] recently reported long-term effects of neonatal care and split suckling provided immediately after birth with consequences on carcass and meat quality characteristics. Furthermore, different responses to neonatal treatments according to birth weight (BW) were observed.

Another early intervention strategy of potential interest is creep feeding (CF), which consists in offering a highly palatable and digestible diet to the suckling piglets [12]. Thus, it ameliorates the weaning transition from milk to solid feed by reducing neophobia at weaning [13,14,15] and facilitating the intestinal microbiome maturation to one able to digest complex nutrients such as fiber or proteins [16,17]. It may also complement the maternal milk production, which could be insufficient for large litters in the last days of lactation [18].

The effectivity of CF on piglet’s growth postweaning is controversial [19]. Creep feeding results are affected by multiple variables, including diet composition, duration of exposure, pellet size, feeder design, and litter size [13,19,20,21]. Although CF is mainly aimed at improving LBW piglets, few studies have specifically compared their response to that of their heavier littermates and or investigated its potential long-term consequences [22]. Moreover, as far as we are aware, there is no available data on long-term effects of CF on carcass or meat quality.

Thus, the aim of the present study was to better understand the effects of creep feeding depending on piglet birth weight on short- and long-term growth, carcass, meat quality characteristics, and microbiome composition. The hypothesis was that creep feeding may produce lasting effects on the microbiome, carcass composition, and meat quality characteristics and that responses may vary according to piglets’ birth weight.

## 2. Materials and Methods

### 2.1. Ethic Statement

The experiment was carried out in March 2021 at a commercial farm from COPISO (Cabanillas, Soria, Spain). Housing conditions complied with European Directive 2008/120/EC and adhered to the provisions of European Directive 2010/63/EC (Article 1, 5.f); there were no invasive procedures or treatments. The Animal Welfare Committee of the Comunidad de Castilla y León reviewed and approved the present study (Ref Fr/bb-2022).

### 2.2. Farrowing and Lactation

Fifty-eight X54 sows (Large White L05 × Landrace L04) from the PIC line (Pic Improvement Company, Sjælan, Denmark) were used in the study. The animals were categorized into two parity classes: first parity (gilts) and second or higher parity (2–3, sows). The animals were inseminated using heterospermic semen from DanBred sires, which was collected and stored under refrigeration for less than 48 h. We obtained 16 litters from the primiparous gilts and 42 from the multiparous sows, with a total of 750 piglets involved in the study.

Sows and gilts were housed in groups of 45 animals in gestation pens during the gestation period. One week before the expected farrowing date, sows and gilts were moved to 2.60 × 2.0 m farrowing pens, with farrowing crates of 1.90 × 0.80 m and slatted floor. All rooms were in the same building. A peripartum diet with 16.5% CP, 2.15 Mcal EN/kg, and 3.95 g SID LYS/Mcal EN was fed at 7.00 h and 15.00 h daily. It was restricted to 3 kg/sow/day from gestation day 112 to day 4 of lactation. From this time point until weaning, sows ate a lactation diet with 16.6% CP, 2.3 Mcal EN/kg, and 3.69 SID LYS/Mcal EN). Feed was provided using a Dositronic^©^ device (Rotecna, Lleida, Spain). Sows had ad libitum access to water. Sows were provided with a lactation diet immediately after farrowing which was progressively increased: 0.5 kg/d until day 11 of lactation, and then 100 g/d in primiparous and 200 g/d in multiparous up to 7.8 kg and 8.0 kg, respectively, until end of lactation. Non-eaten feed was removed.

Piglets were identified with electronic ear tags (MPIG-DATA, Madrid, Spain) and individually weighed. The BW class ranges were established based on mean ± standard deviation (1.32 ± 0.30 kg) as follows: low (<1.02 kg; LBW), normal (1.02–1.62 kg; NBW), and high (>1.62 kg; HBW). One day after farrowing, those piglets exceeding the number of functional teats were distributed to other sows not included in this experiment (13 piglets were used on average per litter), but we maintained similar proportions of BW class within each litter. Weaning was carried out at 25.8 ± 1.9 days. Creep feeding diet (Primefeed Wean Recría, Complementos de piensos compuestos S.A, Navarre, Spain) with 18.1% CP, 2.5 Mcal EN/kg, and 5.53 SID LYS/Mcal EN was offered to piglets (flour form) from day 7 until their weaning in a creep feeder (creep feeding group; CF) in 29 litters (8 primiparous, 21 multiparous). The remaining litters did not receive any feed supplementation for the piglets and were used as the control group. The creep feeder had a J axis, 5 feeding points, and was 25 cm high and 24 cm in diameter (2 L capacity). Creep feeding was offered three times daily. During the first week, each litter received 210 g/day (70 g per feeding). The following week, the daily amount increased progressively from 430 to 630 g, and during the last week, it increased further from 630 to 840 g/day. The bowls were cleaned before each feeding, and any small amount that remained was spread on the heat map. However, this did not guarantee that all the feed offered was consumed, as some of it may have stuck to the animals or ended up on the floor. The remaining 29 litters did not have access to CF (control group; CT). All piglets had ad libitum access to water.

### 2.3. Weaning, Growing, and Fattening

After weaning, piglets were kept in groups of 25 in flat-deck pens of 2.60 × 2.35 m. All replicate pens had similar average body weight. The room temperature for the first week was 25 ± 2 °C and was then reduced 2 °C per week until reaching 22 °C. Water and feed were offered ad libitum with a common feeder of 1.5 m length and 2 water bowl drinkers. Diet and handling were identical to all animals, regardless of previous treatment. Piglets received a weaning diet during the first two weeks after weaning, which had 17.4% CP, 2.52 Mcal EN/kg, and 5.59 g SID LYS/Mcal EN. Then, piglets received a starter diet (17.5% CP, 2.45 Mcal EN/kg, and 5.38 g SID LYS/Mcal EN) during the three following weeks. Piglets were individually weighed at weaning, nursery end (35 days postweaning), and fattening end.

Afterwards, gilts were separated from barrows (as they were intended for reproduction) and were not further studied. Barrows were moved to the growing-finishing farm. There, they were housed in groups of nine pigs in a naturally ventilated finishing barn with 310 × 330 cm pens that had an 80% area of slatted concrete floor.

The feeding program was the same for all barrows and consisted of five different diets based on cereals and soybean designed to meet the changing nutritional requirements during the fattening period. All pigs were slaughtered the same day (at 170–175 days of age).

Dead piglets were recorded daily, and mortality was calculated as the cumulative ratio based on the initial number of piglets participating in the trial (number of dead piglets/initial number of piglets × 100). Mortality was also calculated for each experimental period, using the number of piglets alive at the beginning of each period as the reference. Data are presented as percentages.

### 2.4. Carcass and Meat Quality

Following a 12 h fasting period, 96 barrows (8 per experimental group) were transported 300 km to a commercial abattoir (Incarlopsa, Cuenca, Spain), where they were held in lairage for 5 h. Stunning, slaughtering, and scalding were performed in accordance with European commercial standards. Carcass lean content and the proportions of lean and fat in the hams were assessed using the AutoFom classification system (Carometec Spain, S.L., Barcelona, Spain). Carcasses were individually eviscerated and weighed before the head was removed at the atlanto-occipital joint. The carcasses were then suspended and chilled in a refrigerated room at 2 °C with 90% relative humidity and an airspeed of 1 m/s for 2 h. Subcutaneous fat depth was measured at the P2 point, located approximately 6–8 cm from the midline at the last rib level, and also on the external surface of the ham. Carcass cutting was carried out following the simplified European Community reference method [23]. Loins were kept at 4 °C for 24 h.

*Longissimus dorsi* (LD) muscle samples (200 g each, taken at the level of the last rib) were collected from 82 pigs, with 6 samples representing each experimental group (combinations of 2 treatments, 2 parity categories, and 3 birth weight classes). The samples were placed individually in plastic bags for subsequent analysis.

The moisture, protein, and ash contents of the muscle were determined using a FOSS 6500 Spectrophotometer (Foss Analytical, Barcelona, Spain). Meat color was evaluated with a Chroma Meter (CM-2002, Minolta, Osaka, Japan), which was calibrated beforehand using a standard white tile (CIE, 1976). Measurements were conducted using a D65 illuminant, a 2° observer angle, the SCI mode, and a 1 cm aperture. Five readings per sample were averaged to obtain values for lightness (L*), redness (a*), and yellowness (b*).

Intramuscular fat extraction and methylation were performed following the method described by Segura and Lopez-Bote [24] using freeze-dried samples. The resulting biphasic system was separated via centrifugation (10,000 rpm for 8 min). The solvent was then evaporated under a nitrogen stream, and the remaining lipids were quantified using a gravimetric analysis.

Subcutaneous and intramuscular lipids were esterified by heating at 80 °C for 1 h in a mixture of methanol, toluene, and sulfuric acid (88:10:2, *v*/*v*/*v*). Fatty acids (FAs) were analyzed using gas chromatography (Model 6890, Hewlett Packard, Avondale, PA, USA) equipped with an automatic injector set at 250 °C and a flame ionization detector. The FA methyl esters were separated on a capillary column (HP-INNOWax; 30 m × 0.32 mm × 0.25 μm, Agilent Technologies GmbH, Waldbronn, Germany) under a temperature program ranging from 170 °C to 245 °C. A split injection ratio of 1:50 was applied. Individual FAs were identified by comparison with external standards (Sigma-Aldrich, Madrid, Spain), and their concentrations were expressed as percentages of total fatty acids.

### 2.5. Rectal Microbiome Extraction

Sixty-nine piglets were selected for the microbiome analysis (thirty-five from the control group and thirty-four from the CF group). Within the control group there were 9 HBW, 7 LBW, and 19 NBW piglets. In the CF group, there were 7 HBW, 9 LBW, and 18 NBW. Feces samples from the rectum were obtained at 3 time points from those 69 piglets: three days prior weaning (T1), 7 days after weaning (T2), and before the slaughtering of the pigs (T3). Sampling was performed with sterile swabs (Copan Diagnostics, Murrieta, CA, USA), introduced in the rectum and rotated. After sampling, the edge of the swab was cut and introduced in a 2 mL tube prefilled with 1 mL of preserving solution (DNA/RNA Shield, Zymo Research, Irvine, CA, USA), closed and transferred to the lab at room temperature and then stored at −20 °C until use.

For each sample, DNA was isolated from the swabs using the ZymoBIOMICS DNA Miniprep Kit (Zymo Research, USA) following the manufacturer’s instructions. The 16S rRNA gene, specifically the V3–V4 hypervariable region, was amplified and sequenced using the Illumina MiSeq^®^ paired-end platform (Illumina, San Diego, CA, USA). Sequencing was performed by an external provider (IPBLN-CSIC, Granada, Spain). The primers utilized for the amplification process were S-D-Bact-0341-b-S-17 and S-D-Bact-0785-a-A-21 [25], which produce amplicons of 464 bp.

Bioinformatic analyses of raw fastq archives and their processing to Amplicon Sequence Variants (ASVs) were performed using the DADA2 1.28.0 algorithm [26]. Taxonomy was studied using SILVA database 138 (https://www.arb-silva.de/documentation/release-138/ (accessed on 10 October 2023).

### 2.6. Statistical Analysis

#### 2.6.1. Farm, Carcass, and Meat Quality Data

The statistical analysis was performed using SAS software version 9.4 (SAS Institute Inc., Cary, NC, USA). Mortality data were evaluated using the chi-square test. Normality and homogeneity of variances were assessed through the Shapiro–Wilk test and Levene’s test, respectively. For overall reproductive data, the sow was the experimental unit. To study differences in weight and gains, as well as carcass and meat quality of the progeny, a completely randomized design was carried out using the mixed model contained in SAS. The experimental unit in this case was the piglet, and the sow was included as a random effect in the statistical model. The number of pigs, carcasses, or meat samples per treatment is included in the tables. For live weight and gains data, this refers to the initial number of animals. Fixed effects for weight and gains were the creep feeding treatment, parity, and sex. Birth weight class was excluded as a fixed effect due to the non-normal distribution of the data. Alternatively, the impact of birth weight was assessed through a linear regression analysis, and differences in slopes were evaluated using Student’s *t*-test. In all cases, carcass and meat quality traits adhered to a normal distribution. Consequently, treatment, parity, and BW categories were incorporated as fixed effects in the statistical analysis. For carcass and meat quality data, carcass weight was included as covariable in the model to ensure that observed differences were not confounded by carcass weight. Data are presented as LS means and pooled standard deviation (SD). When *p* < 0.05, differences between treatment means and interactions were considered statistically significant, while values of 0.05 < *p* ≤ 0.10 was considered marginally significant.

#### 2.6.2. Microbiome Data

R version 4.3.1. [27] was used to analyze the microbiome from rectal samples. The α- and β-diversities studies in the 69 samples comparing treatments, BW, and time of sampling were performed with the vegan R package version 2.6-4 after rarefaction [28]. Due to the strong time effect found in alpha and beta diversities, we subsampled the animals to analyze separately each time point in both analyses. Plots to visualize diversity results were created with *phyloseq* 1.44.0 and *ggplot2* 3.4.4 [29]. Alpha-diversity measures (observed richness and Shannon index) were calculated using the function *estimate_richness* from *phyloseq*. Statistical significance was assessed with an ANOVA including the animal as a random effect, and treatment, BW, and time of sample collection as fixed effects and their corresponding interactions.

The Bray–Curtis dissimilarity index was computed using the phyloseq’s distance function and employed to analyze β diversity, in combination with a Principal Coordinate Analysis (PCoA) plot. To evaluate statistical significance, a PERMANOVA was conducted using the adonis2 function within the vegan package. The model included birth weight adjusted for time point and the interaction between birth weight and time point. Piglet identity was incorporated as a random effect. Results were considered statistically significant at *p* < 0.05. A differential abundance analysis was conducted using the linDA method, following the exclusion of samples with fewer than 30,000 reads. Linear models were applied to identify significant differences [30] at the genus level. The model included the BW and time-point effects, as well as the piglet as a random effect. As in diversity analyses, time points were studied separately to avoid any time effect. Significance was considered with a q-value < 0.05.

## 3. Results

### 3.1. Mortality

The effect of BW, treatment, parity, and sex on mortality during the first week of life, and the accumulative value along the whole lactation period and the first week postweaning is shown in Table 1. Most mortality was concentrated during the first week of life in the LBW piglets. The LBW piglets showed a value nearly six-fold higher than that of the HBW piglets. No effect of treatment, parity, or sex on mortality was observed in any case.

### 3.2. Weight and Gains

The farrowing rate and the number of total born, born alive, and stillborn piglets were 89.1%, 17.4, 15.7, and 1.7, respectively, while the number of mummified piglets was 1.8, with no differences between treatments. The feed intake of primiparous and multiparous sows on the farrowing day was 2.8 ± 0.63 and 3.1 ± 0.60 kg, respectively. Actual mean feed intake along lactation was 129 ± 11.3 and 156 ± 14.9 kg/sow for primiparous and multiparous sows, respectively, with no effect of the creep feeding treatment.

The effect of CF treatment, parity, and sex on weights and gains is shown in Table 2. Piglets from multiparous sows were heavier, especially at early ages (*p* < 0.01 and *p* < 0.02 for BW and weaning weight, respectively). No effect of CF or interactions was observed in any case.

**Figure 1:**
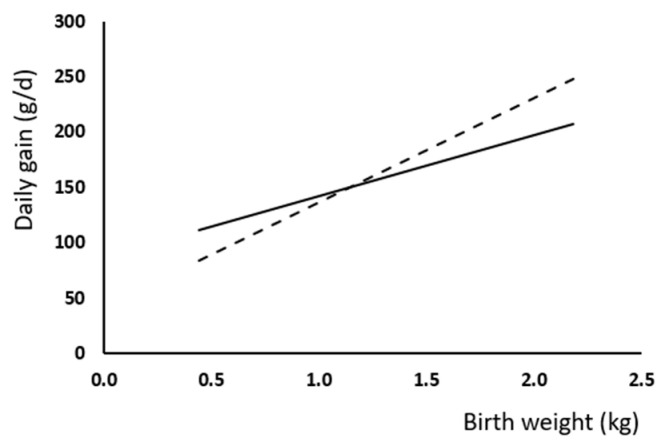
Relationship between birth weight (BW) and daily weight gain (g/d) along lactation because of the presence (CF, dotted line) or absence (Ctr, filled line) of creep feeding. Daily gain in CT group = 87.5 (±19.7) + 54.9 (±13.7) × BW (kg); *p* < 0.0001; R^2^ = 0.16; daily gain in CF group (g/d) = 42.5 (±14.3) + 94.1 (±10.5) × BW; *p* < 0.0001; R^2^ = 0.25. Slopes were different (*p* < 0.05).

A regression analysis was applied to evaluate the influence of the BW and CF treatment on live weight and weight gain. Linear regression provided the best fit to the data, revealing distinct responses in daily weight gain during lactation, with the heavier piglets showing higher gains (*p* < 0.001) (Figure 1). Moreover, differences between slopes indicates a better response to the CF treatment of heavier piglets, while a possible detrimental effect on lactation in low-BW animals. Specifically, we found that a 100 g difference in birth weight resulted in a variation of approximately 12–13 g in daily weight gain during lactation (Figure 1). However, it should also be noted that the R^2^ value was low in both cases.

### 3.3. Carcass and Meat Quality Characteristics

The effect of BW, CF treatment, and parity on slaughter and carcass weight, as well as carcass and meat quality characteristics are presented in Table 3 and Table 4, respectively. A marked effect of BW was observed on slaughter and carcass weight, but no effect of BW, CF, or parity was observed on carcass characteristics (Table 3) when carcass weight was used as co-variable. A higher quantity of intramuscular fat was observed in LBW barrows (*p* < 0.009) and in those animals coming from multiparous sows (*p* < 0.044). Neither the effect of CF on meat quality characteristics nor interactions were detected in any case.

### 3.4. Microbiome Analyses

#### 3.4.1. Short-Term Effects

Microbiome analyses were performed in fecal samples from the rectum and consisted in the study of phyla and genera composition, diversity (alpha and beta), and differential abundance.

The most abundant phyla before and after weaning were *Firmicutes* and *Bacteroidetes*. The most abundant genera were *Bacteroides* and *Escherichia*/*Shigella* before weaning and *Prevotella* and *Bacteroides* after weaning.

Alpha-diversity indexes were higher after weaning (*p* < 0.001 for observed richness and Shannon index). However, CF treatment and BW did not affect the alpha diversity of the piglets.

**Figure 2:**
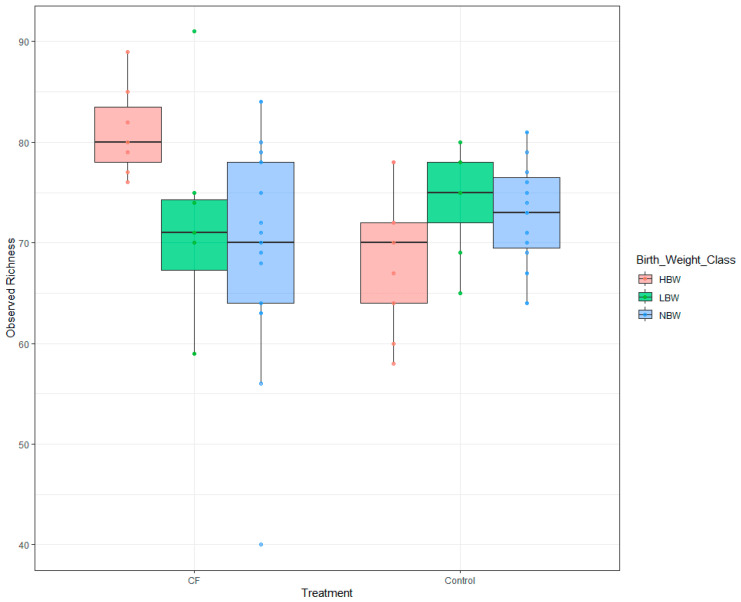
Observed richness according to treatment and birth weight after weaning. CF = creep feeding. Low: weight equal to or less than 1.02 kg; normal: weight between 1.02 and 1.62 kg; high: weight over 1.62 kg.

To avoid a time effect, we analyzed the samples taken three days before weaning and the ones taken a week after weaning separately. No differences were found before weaning, but after weaning, there was a significant interaction between birth weight and CF treatment (*p* < 0.05). Thus, HBW piglets that ate creep feeding had higher observed richness than control HBW piglets, whereas NBW and LBW piglets in both control and CF groups showed similar observed richness values (Figure 2).

**Figure 3:**
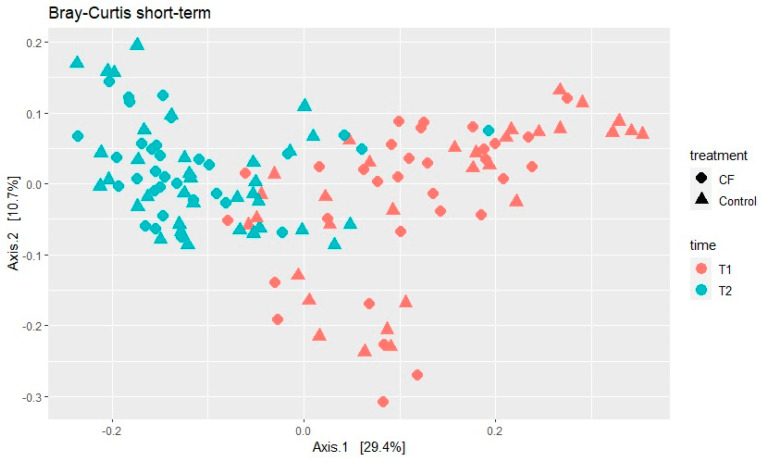
Treatment and time effects in Bray–Curtis’s beta diversity dissimilarity index in rectal samples from pigs 3 days before weaning (T1) and a week after weaning (T2). CF = creep feeding.

The beta diversity was analyzed using the Bray–Curtis dissimilarity index. During the short-term developmental period, no significant differences were found between treatments nor BW. Only time had significant effects on the beta-diversity index (*p* < 0.001, Figure 3).

LinDA [30] was used to study differences between treatments, BW groups, and time points. No differences were found between control pigs and those that underwent a CF treatment, nor between BW groups. Time was the most important factor in determining differences in genera abundance. Thus, we found 93 differences in the genera composition between piglets 3 days prior weaning and day 7 after weaning. Genera from the *Prevotellaceae* family such as *Prevotellaceae_NK3B31*_group or *Prevotellaceae_UCG-003* increased after weaning, whereas genera such as the Lactobacillus decreased after weaning. Afterwards, each time point was studied separately again with no significant differences between treatments nor BW.

#### 3.4.2. Long-Term Effects

In the rectal samples obtained before slaughtering, the two most abundant phyla were *Firmicutes* and *Bacteroidetes* and the most abundant genera were *Clostridium* and *Prevotella*.

**Figure 4:**
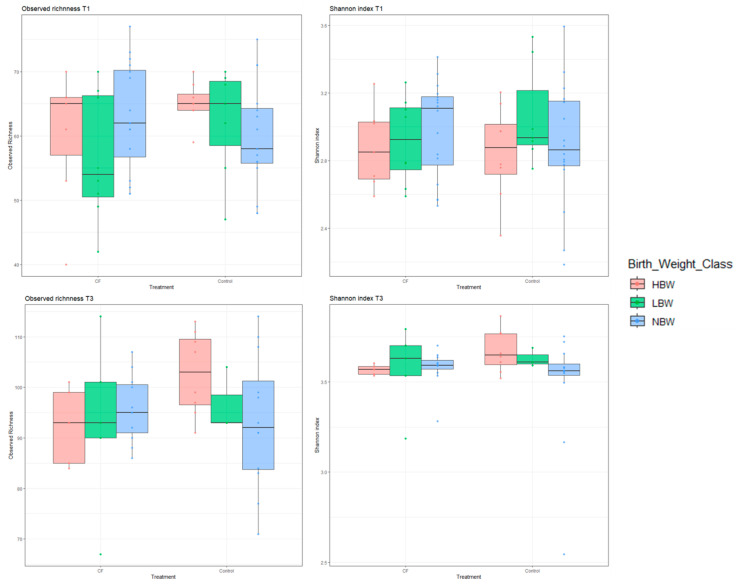
Alpha-diversity analyses (observed richness and Shannon index) three days prior weaning (T1) and before slaughtering (T3) in pigs that underwent creep feeding treatment (CF) or not (control) separated by birth weight (low: weight equal to or less than 1.02 kg; normal: weight between 1.02 and 1.62 kg; high: weight over 1.62 kg).

Microbiome composition changed with the age of the animals similarly in all treatment and BW groups. Thus, observed richness and Shannon index were significantly higher in animals before slaughtering when compared to the indexes before weaning (*p* < 0.001 for both; Figure 4). Thus, eating the creep feed increased the number of different species found in the feces, indicating changes from a microbiota more specialized in milk digestion to a more mature composition. Based on this observation, it seems that the high-birth-weight piglets were the ones that ate more creep feed and therefore benefitted more from it.

**Figure 5:**
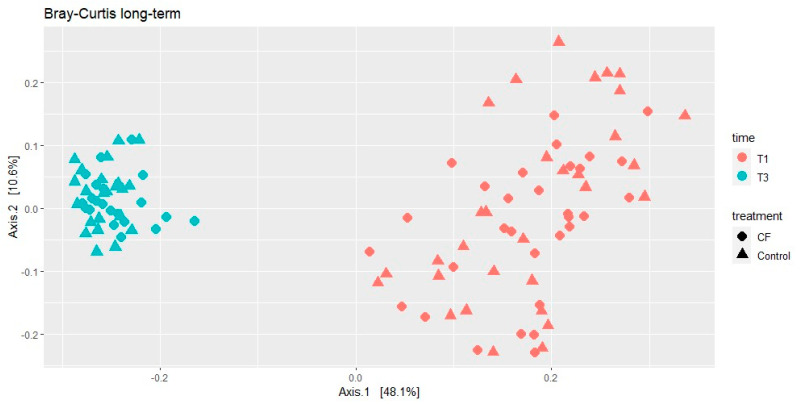
Time effect in Bray–Curtis beta diversity dissimilarity index in rectal samples from pigs 3 days before weaning (T1) and before slaughtering (T3).

Bray–Curtis dissimilarity indexes clustered samples in two groups according to the time of sampling (Figure 5). Regarding the differential abundance analysis, 123 genera were differentially abundant between T1 and T3 ages. *Butyricimonas* was the genus more overabundant in T1, whereas at T3, the more overabundant genus was *Terrisporobacter*.

## 4. Discussion

In this study, mortality during lactation was affected by BW. Thus, LBW piglets showed a six-fold higher mortality than normal-weight piglets. This effect was mainly concentrated in the first days after birth, which agrees with previous observations [31], due to differences in body fat or glycogen reserves [32,33]. Low birth weight also had consequences on growth rate at later life stages. Thus, a 100 g difference in live weight at birth resulted in approximately 12–13 g lower daily weight gain during lactation and a difference of about 2 kg in live weight at slaughter. These findings are consistent with the previous literature [1,10,34].

To counteract this situation, especially in the context of hyperprolific sows, different strategies are being tested, such as CF. Interestingly, even if CF at farms usually aims to boost LBW piglets’ development, there are scarce data on the effect of CF depending on BW. Wolter et al. [35] analyzed the effect of CF by BW by separating piglets in high vs. low BW groups. Similarly, Huting et al. [22] analyzed the differences in both uniform or mixed BW litters according to CF treatment and BW. However, both experiments were conducted in experimental farms. Thus, more research is needed in commercial farm conditions to fully understand the effect of CF depending on the BW of the piglets.

In our study, CF showed no effect on piglet mortality. This was an expected result since most piglet mortality took place before the CF supplementation (first 48 h). In fact, previous data have shown that human intervention in early days may negatively affect sow–piglet interaction and be counter-productive [10].

Creep feeding affected lactation ADWG in different ways depending on the piglets’ BW. Heavier piglets showed higher weight gain when CF was provided, while CF reduced growth potential in light piglets in accordance with previous research [22,35]. Thus, high-BW pigs could benefit from a complementary source of nutrients for their high growth potential. This explanation would also agree with the results of Huting et al. [22], in which heavy piglets consumed more CF when allocated with other high-BW piglets than in mixed litters. However, low-BW piglets showed a lower response to the CF diet. It could be hypothesized that CF practice may increase social competition with heavy-BW piglets. Furthermore, one of the most important factors affecting CF consumption are related to the CF diet itself, such as its form or the feed complexity [14,15,19,36]. Thus, another possibility is that the CF diet is too complex for the unmatured digestive system of low-BW piglets, that have low trypsin and lipase activity [37].

The differential consumption of CF according to BW also had consequences for the microbiome composition, with HBW piglets having higher alpha diversity than low and normal BW piglets on day seven after lactation. Alpha diversity is linked to gut health, since a high alpha diversity implicates a strong stability and adaptability to different feeding resources [38]. However, most microbiome differences were due to the time of sample collection (and, therefore, age of the animal), being potentially related to all the changes in the environment and diet. Weaning had important effects on microbiome composition. This is in accordance with the previous literature reflecting the major effects that the change in feeding (from maternal milk to solid feed) have on microbiota composition [39]. It is known that the change from maternal milk to solid feed, with higher carbohydrates content, provokes a change from a microbiota rich in *Bacteroides* and *Proteobacteria* to an increase in *Prevotella*, *Succinivibrio* or *Roseburia* [39,40,41,42]. CF has been previously studied to ameliorate this microbiota transition, avoiding dysbiosis and diarrhea. In fact, preweaning diet has previously been shown to greatly change the microbiome composition, increasing the abundance of genera implicated in the digestion of complex feed [17]. However, the preweaning diet composition of this diet was different to ours, with high levels of soluble and insoluble fiber [17]. Limited results were obtained when using a more standard CF diet [43], similarly to the lack of effect of the CF diet in the present study.

Few research studies have addressed the long-term consequences of CF, with most of them focusing on lactation and nursery periods [44]. Previous data have shown effects of CF long after weaning, with increased feed intake pattern [45]. However, in accordance with previous results, CF did not have an impact on pigs’ growth up to slaughter [13,36,45,46]. In our experiment, CF did not affect meat quality nor carcass characteristics. However, to the best of our knowledge, there are no previous data on the CF effect on these parameters, so no comparison with other studies can be made. In this experiment, we observed higher concentration of IMF in LBW piglets in the LD muscle, which agrees with previous observations [11]. This study was conducted in a commercial production environment, where certain factors, such as the sire effect, were not entirely controlled due to the use of heterospermic insemination. According to our results, the hypothesis that creep feeding may have lasting effects on the microbiome, carcass composition, and meat quality characteristics is not supported. It is interesting to note that other early-life interventions, such as neonatal care (including tying the umbilical cord, clearing mucus and debris from the nasal and oral cavities, and gently guiding the mouth onto a functional teat.) or split suckling increases lean content, especially in LBW piglets [10,47]. The increase in lean content could be derived from the metabolic stimulation and neonatal imprinting effect of colostrum, which could regulate metabolic processes like insulin sensitivity, adiposity, redox reactions, or lipogenic activity, among others [48]. Thus, the lack of long-term response of creep feeding suggests that interventions to ameliorate viability and produce long-term effects should focus on the very first initial stages of life.

## 5. Conclusions

Unlike other neonatal intervention practices (such as early neonatal care and split suckling), we did not observe any long-term effect of CF on the microbiome, growth, carcass, or meat quality. The short-term effects of CF appeared to be most pronounced during lactation and possibly the early postweaning. These short-term effects were driven by BW, with HBW piglets benefiting the most from creep-feeding practices.

## Figures and Tables

**Table 1:** Effect of birth weight (BW), creep feeding treatment (T), parity (P), and sex (S) on mortality (%) from birth to 7 days of life (before treatment), from start of treatment to weaning (CF to weaning), and from weaning to one week after weaning (weaning + 7 d). Data are presented per period and cumulatively and were analyzed by Chi-square comparison.

	Birth Weight (BW)			Treatment (T)		Parity (P)		Sex (S)		*p*-Value			
	LBW	NBW	HBW	Ctr	CF	PP	MP	F	CM	BW	T	P	S
Birth to 7 days	36.4	9.6	5.6	14.0	12.0	10.9	14.1	11.1	14.5	0.001	0.51	0.34	0.28
CF to weaning	4.1	2.8	0.0	1.6	3.7	3.3	2.1	2.5	2.3	0.062	0.11	0.47	0.86
Weaning + 7 d	0.0	2.2	1.2	1.7	1.7	1.7	1.7	1.7	1.7	0.453	0.97	0.96	0.98
Cumulative mortality from birth													
Weaning	39.0	12.5	5.6	15.2	15.9	14.5	15.9	13.1	17.0	0.001	0.95	0.45	0.33
Weaning + 7 d	39.0	14.1	6.7	16.7	16.8	15.2	17.4	14.6	18.1	0.001	0.96	0.57	0.32

LBW: birth weight equal to or less than 1.02 kg (n = 182); NBW: birth weight between 1.02 and 1.62 kg (n = 446); HBW: birth weight over 1.62 kg (n = 130); Ctr: control (n = 368); CF: creep feeding (n = 382); PP: primiparous (n = 202); MP: multiparous (n = 548); F: female (n = 366); CM: Castrated male (n = 384).

**Table 2:** Effect of creep feeding provided during lactation on weight and gain during lactation and nursery (35 days postweaning).

	Treatment (T)		Parity (P)		Sex (S)		SD	*p*-Value		
	Ctr	CF	PP	MP	F	CM		T	P	S
Birth weight (kg)	1.37	1.30	1.21	1.38	1.33	1.34	0.36	0.269	0.010	0.224
Weaning weight (kg)	5.72	5.69	5.18	5.90	5.72	5.70	1.48	0.836	0.021	0.377
Nursery end weight (kg)	29.20	28.14	26.92	29.49	23.18	29.27	6.77	0.706	0.073	0.003
ADG lactation (g/d)	164.73	165.30	152.60	169.59	164.98	165.07	49.5	0.868	0.091	0.421
ADG nursery (g/d)	674.10	666.17	619.79	697.94	521.86	685.93	164.6	0.869	0.066	0.004

Ctr: control (n = 368); CF: creep feeding (n = 382); PP: primiparous (n = 202); MP: multiparous (n = 552); F: female (n = 356); CM: castrated male (n = 384); SD: standard deviation. The mean birth, weaning, and end-of-nursery weights according to BW class were 0.9 ± 0.13, 1.3 ± 0.16, and 1.8 ± 0.12 kg; 4.3 ± 1.03, 5.6 ± 1.30, and 6.8 ± 1.46 kg; and 21.9 ± 4.02, 28.2 ± 5.92, and 33.9 ± 6.06 kg, respectively. There were no interactions across any of the measured criteria, *p* > 0.10.

**Table 3:** Effect of birth weight (BW), creep feeding treatment (T), and parity (P) on barrows’ mean slaughter weight, carcass weight, and carcass yield, as well as LS means of barrows’ carcass characteristics.

	Birth Weight (BW)			Treatment (T)		Parity (P)		SD	*p*-Value			
	LBW	NBW	HBW	Ctr	CF	PP	MP		BW	T	P	Cov-CW
Slaughter weight (kg)	118.3 ^a^	131.1 ^b^	140.9 ^c^	130.5	130	123.8	132.8	12.3	0.001	0.304	0.058	--
Carcass weight (kg)	92.7 ^a^	100.5 ^b^	107.7 ^c^	100.4	99.7	97.6	101.1	11.5	0.001	0.255	0.495	--
Carcass yield (%)	77.2	77.1	76.7	77.4	76.7	77.8	76.7	7.7	0.859	0.875	0.713	--
Subcutaneous fat												
P2 (mm)	21.45	20.67	20.18	20.78	20.76	20.25	21.28	3.43	0.662	0.978	0.28	0.001
Ham (mm)	15.61	14.99	14.25	15.03	14.87	14.89	15.01	2.39	0.386	0.819	0.861	0.001
Lean (%)	55.85	56.97	57.19	56.71	56.63	57.1	56.24	2.89	0.432	0.914	0.285	0.001

LBW: birth weight equal to or less than 1.02 kg (n = 32); NBW: birth weight between 1.02 and 1.62 kg (n = 32); HBW: birth weight over 1.62 kg (n = 32); Ctr: control (n = 48); CF: creep feeding (n = 48); PP: primiparous (n = 48); MP: multiparous (n = 48); SD: standard deviation; Cov-CW: co-variable carcass weight. There were no interactions across any of the measured criteria *p* > 0.10. Different superscripts in mean values between BW classes indicate significant differences (*p* < 0.05).

**Table 4:** Effect of birth weight (BW), creep feeding treatment (T), and parity (P) on the chemical composition (%) of barrows’ longissimus dorsi and subcutaneous fat, as well as pH and color values.

		Birth Weight (BW)			Treatment (T)		Parity (P)		SD	*p*-Value			
		LBW	NBW	HBW	Ctr	CF	PP	MP		BW	T	P	Cov-CW
	IMF	2.18 ^a^	1.83 ^b^	1.63 ^b^	1.80	1.96	1.74	2.02	0.47	0.009	0.245	0.044	0.01
	Moisture	74.13 ^b^	74.29 ^ab^	74.67 ^a^	74.43	74.30	74.51	74.22	0.50	0.038	0.388	0.049	0.00
	Protein	23.03	23.24	23.12	23.18	23.08	23.15	23.11	0.37	0.163	0.397	0.751	0.14
	Ash	1.74	1.76	1.72	1.74	1.74	1.75	1.74	0.05	0.050	0.973	0.620	0.24
	pHu	5.69	5.67	5.66	5.68	5.67	5.67	5.68	0.11	0.827	0.606	0.668	0.00
	L value	58.61	58.41	59.34	58.67	58.90	58.84	58.74	2.31	0.556	0.729	0.883	0.00
	a value	2.57	2.59	2.30	2.41	2.57	2.49	2.49	0.52	0.324	0.310	0.998	0.37
	b value	13.80	13.75	13.69	13.64	13.86	13.76	13.73	0.83	0.954	0.350	0.896	0.03
Fatty acids in subcutaneous fat													
	C16:0	23.59	23.20	23.10	23.36	23.23	23.05	23.54	0.90	0.284	0.596	0.062	0.03
	C18:0	12.59	12.56	12.68	12.76	12.46	12.56	12.66	0.64	0.861	0.115	0.599	0.12
	C18:1n-9	38.72	38.57	37.63	38.06	38.55	37.78	38.83	1.99	0.371	0.398	0.070	0.61
	C18:2n-6	10.90	11.36	12.20	11.55	11.42	12.01	10.96	1.88	0.242	0.817	0.057	0.08
Fatty acids in intramuscular fat													
	C16:0	23.58	23.12	22.95	23.29	23.14	23.28	23.15	1.07	0.259	0.628	0.662	0.01
	C18:0	12.37	12.42	12.85	12.65	12.44	12.71	12.38	1.28	0.611	0.566	0.366	0.11
	C18:1n-9	41.28	40.53	41.00	41.14	40.73	41.19	40.69	4.87	0.867	0.768	0.722	0.63
	C18:2n-6	12.63	13.24	13.50	13.03	13.22	12.92	13.33	1.48	0.266	0.658	0.335	0.04

LBW: birth weight equal to or less than 1.02 kg (n = 32); NBW: birth weight between 1.02 and 1.62 kg (n = 32); HBW: birth weight over 1.62 kg (n = 32); Ctr: control (n = 48); CF: creep feeding (n = 48); PP: primiparous (n = 48); MP: multiparous (n = 48); Cov-CW: co-variable carcass weight; IMF: intramuscular fat; SD: standard deviation. There were no interactions across any of the measured criteria *p* > 0.10. Different superscripts in mean values between BW classes indicate significant differences (*p* < 0.05).

## Data Availability

Data are contained within the article.
